# Chicago Gynæcological Society; Meeting August 20

**Published:** 1886-11

**Authors:** 

**Affiliations:** 2330 Indiana Avenue


CHICAGO GYNÆCOLOGICAL SOCIETY.

*Regular Meeting, Friday August 20, 1886.*—THE VICE-PRESIDENT, HENRY T. BYFORD, M.D., in the chair.

I.—DR. W. W. JAGGARD exhibited

AN OVUM CORRESPONDING TO THE FOURTEENTH WEEK OF PREGNANCY, SHOWING TWIN PREGNANCY, WITH ONE PLACENTA, ONE CHORION, ONE AMNION, BOTH EMBRYOS OF THE MALE SEX.

The interesting specimen was placed at his disposal through the courtesy of Dr. Daniel H. Williams, of Chicago. The egg corresponded to the fourteenth week of pregnancy. It was a case of twin pregnancy, with one placenta, one chorion and one amnion. The embryos were equally well developed and were of the male sex.

The case illustrated one of the modes of origin of multiple pregnancy. An ovum may have two nuclei, and an embryo may be produced from each nucleus. Under these conditions, the fecundated ovum has one placenta (or there is anastomotic communication between two fused placentæ), one chorion and two amnions. The amniotic septum may be broken down or absorbed, and the embryos may be contained in a single amniotic sac, as in the specimen exhibited.

In a case of single placenta, or fused placentæ with anastomotic communication, and a single chorion, the twins are always of the same sex. (Hyrtl, Spaeth, Braun.)

II.—Discussion of PROFESSOR JOHN BARTLETT'S paper, entitled

A PROPOSED MODIFICATION OF PORRO'S OPERATION.

(Professor Bartlett's paper appeared in the September number of this journal.)

PROFESSOR A. REEVES JACKSON said: I have never performed Porro's operation, and am not sufficiently familiar with the literature of the subject to be a proper person to open or even take part in the discussion. I confess I scarcely understand what advantages this operation proposed by the essayist offers over the improved operation by Sänger. I would like to know whether Dr. Bartlett has performed this operation either upon the living subject or the cadaver. It seems to me there are practical difficulties in the way. In a review by Harris, of Philadelphia, in the *American Journal of the Medical Sciences*, of the work of Mangiagalli "On the More Recent Modifications of the Cæsarean Section," it is stated that it had been proposed to invert the uterus; for the purpose, however, of lessening the danger from septic infection, and not to facilitate the amputation, as is designed by the suggestion of Dr. Bartlett.

DR. E. J. DOERING asked how often the Cæsarean operation had been performed in Chicago.

PROFESSOR W. W. JAGGARD thought Dr. Bartlett's paper a very ingenious essay, although not based upon sound surgical principles. In the first place, he thought the title of the essay a misnomer. The operative procedure proposed by Dr. Bartlett was not in any sense of the term a modification of or a substitute for Porro's operation. It was a perfectly distinct operation. Dr. Bartlett's method offered no advantages over Porro's operation, as modified by Müller and others. The abdominal cavity is not more thoroughly closed. The presence of a large pedicle does not embarrass the closure of the abdominal incision. The relation of the parts in the suggested procedure are not more natural and much less strained than in the *status* in which Porro's method leaves them. Drainage is entirely unnecessary when Porro's operation has been skillfully performed.

On the other hand, the positive disadvantages are numerous: The dangers of shock and hæmorrhage in artificial inversion of the uterus has been very much underestimated by Dr. Bartlett. The cases, collected from the literature of the subject, when they were at all relevant, were questionable as to authenticity. Accidents occurring to the uterus among the lower animals could not be adduced in evidence as to what would be the probable effect upon human beings under similar conditions. The thermo-cautery was inadequate to the arrest of hæmorrhage from a large incision through the walls of the pregnant uterus.

The uterus could only be inverted with ease, when it was pathologically flaccid,—an exceptional condition. Porro's operation was performed in cases of the simple, flat rhachitic pelvis, when the antero-posterior diameter of the brim was 6 cm. or under. Above 6 cm. craniotomy or the forceps is indicated. It would be very difficult to invert the uterus through the conjugate, oblique or transverse diameter under such conditions. In the pelvis of Robert, or in the osteomalacic pelvis, in which the degree of contraction is usually higher, artificial inversion of the uterus would be well nigh impossible.

Then amputation of the inverted uterus is a dangerous operation *per se*. Of the 48 cases collected by Dr. West,* 12 terminated fatally. Of 58 cases of amputation of the inverted uterus, reported from a German source,† 18 terminated

---

* Diseases of Women, p. 240.

† American Journal of Obstetrics, Aug., 1868.

fatally. "In 106 cases of amputation by ligature* and otherwise, over 31 per cent of deaths occurred." But it is not necessary to multiply statistics. So great is the mortality of this operation, that A. Martin† has proposed as a substitute the total extirpation of the uterus.

If, then, upon *à priori* grounds Dr. Bartlett's suggestion has no real advantages over the modified Porro operation, and, on the other hand, possesses actual disadvantages, it is scarcely probable that the expedient will receive serious consideration.

DR. J. SUYDAM KNOX said: Dr. Jaggard has about covered the objections I intended to make. My impression is that Dr. Bartlett in his paper has over-estimated the relaxation of the uterus immediately after delivery, and the ease with which inversion can be accomplished. Atony of the uterus is the first cause of inversion; and when we consider how minute is the per-centage of inversions in the vast number of labors, we can fairly assume that relaxation immediately after delivery seldom occurs. If this be so, inversion, even with the *vis a tergo*, would be extremely difficult. Again atony of the uterus is the cause of the most dangerous symptom or complication of inversion, namely, hæmorrhage; therefore the cases most favorable for the operation of Dr. Bartlett would be the last ones in which so doubtful an experiment would be tried. The Doctor has made a valuable suggestion. Any method that successfully removes the uterine stump from the abdominal cavity, without attaching it to the abdominal incision, advances the operation of hysterectomy. In the ablation of the non-pregnant uterus, I think Dr. Bartlett's method finds its best opportunity.

---

* Emmet: Principles and Practice of Gynecology, 1884, p. 436.

† Pathologie und Therapie der Frauenkrankheiten, 1885, p. 144.

PROFESSOR JAMES H. ETHERIDGE asked if the performance of inversion by forcible traction involved the full dilatation of the neck of the uterus. How does Dr. Bartlett propose to accomplish this, does he dilate it forcibly? With the uterus well up beyond the umbilicus, how do the broad ligaments come out of the pelvis, and with the uterus forced clear down out of the vulva, how much traction is there going to be on these broad ligaments? Is there room enough to permit the uterus to be drawn down?

Why, under the circumstances, could not forceps be immediately applied to the edge of the cut uterus, and arrest the hæmorrhage, and the work be then proceeded with at pleasure? I speak of hæmostatic forceps.

DR. E. W. SAWYER said: It seems a little presumptuous for one who has never had experience in this department to attempt to enlighten the Society. One of the most interesting questions to be decided is which operation to perform. I confess if I were confronted to-night with one of these cases I would be wholly incompetent to decide between the Cæsarean operation and the operation of Porro. It may be interesting to read the words of Lawson Tait upon this very point, showing his preference for the new operation, so-called. In the fifth number of the *British Gynæcological Journal*, he says: "The whole of my experience in meddling with the pregnant uterus by abdominal section, consists of five cases, three of the ordinary Cæsarean section and the two I am about to describe in detail. Of the Cæsarean sections one was performed for malignant disease of the vagina about fourteen years ago, the other two for deformed pelvis, respectively seven and five years ago, and the mothers died, and only one of the children is now living. The results indeed are such as to determine me never to repeat this procedure, having before me the arguments of Dr. Godson and the fact that both my amputation cases have recovered." At the same meeting Dr. Routh said: "That he was much interested and instructed by Dr. Lawson Tait's paper. At the same time he could not help making some criticisms upon it. First, he believed that Mr. Tait had exaggerated the mortality of the Cæsarean section. It was not anything like 99.971 per cent. Churchill stated that out of eighty cases twenty-three mothers were saved, or 28.7 per cent., forty-four children being saved. Dr. Radford, out of seventy-six cases he collected, found 14.28 were saved, and forty-six children were also saved. Dr. West, out of 409 cases, states the recoveries as 38.4 per cent., 237 children being saved. Now he (Dr. Routh) could not help feeling that if in these days of improved antiseptic abdominal surgery, the same skill and care were taken in cases of Cæsarean section, the safety of the mother would be much more common." It is interesting to see how gentlemen will differ in their opinions upon such an important thing as the selection of an operation in an emergency case. So I am still in doubt whether to adopt the modern method of Porro or to depend upon the Cæsarean section, which the remarks of Dr. Routh would indicate is quite as favorable.

At the request of Dr. Etheridge, Dr. Sawyer narrated the following case, showing the shock and hæmorrhage of acute inversion. I will state very briefly an experience which, no matter how long I may live, seems as if it would never become dim. I have never had any doubt that the determining cause of the acute inversion in this case was the enormous distension of the uterus due to the large quantity of *liquor*

*amnii.* Before the woman was delivered, I was impressed with the fact that she probably had twins, but this was not the case. When the woman was delivered the bed was flooded, the *liquor amnii* flooding the room even. I put my hand upon the woman's belly, as is my custom, and at the first indication of contraction of the uterus, I substituted the husband's hand for mine that I might pay attention to the child. I am confident that the husband's fingers dimpled that uterus. I had no sooner detached the child than I gave the usual teaspoonful of ergot. I was in a hurry, on account of the flabby condition of the uterus, and for fifteen minutes my time was occupied in paying attention to the child, getting it to breathe. The woman, who had recovered from a small quantity of ether which I gave her, threw up her hands and I saw she was pale. I put my hand under her husband's and felt the edge of the uterus like the edge of a saucer, I could define the margin of the crater, my finger in the vagina met the globe inverted and the truth flashed across me that I had an inverted uterus. Now fifteen minutes had not elapsed before that uterus was so firmly ergotized that it was impossible to replace it. I immediately resumed ether and the woman began to snore, but that made no difference, the womb was ergotized and the woman died from shock and hæmorrhage with the uterus unreduced.

Dr. Jaggard has called attention to the enormous hæmorrhage, and this reminds me of a case in which I removed a fœtus from the abdomen of a woman in the little town of Boulder. The fœtus had been in the uterus for three and a half years. It was an adventitious uterus, the exact structure of which could not be ascertained, but the hæmorrhage from the false uterus was enormous, and, I think, destroyed the woman. If the false uterus and adventitious sac could bleed to that degree and so early in pregnancy, the dangers of hæmorrhage must surely be greater in the uterus at term containing a living fœtus and an active placenta.

This operation was done in 1874. The hæmorrhage was cavernous. We arrested the hæmorrhage by seizing the edges and puckering them up and tying an enormous ligature around the stump. For a moment that arrested it, but the woman subsequently died.

DR. H. T. BYFORD said: Like any other operation, this one, supposing it to be an operation that has been performed, has its limitations. I think Dr. Jaggard's suggestion that a greatly contracted pelvis might afford sufficient difficulty to make the operation impracticable, is a good one, although I think that the uterus might be inverted through a pelvis too small or too much distorted for a safe craniotomy. Another limitation would be an undilated condition of the cervix. The irritation produced by rapid dilatation would certainly render the cervix unfit to be left as a stump, and make the Cæsarean or Porro operation preferable. If the os is already dilated, then Thomas' revised laparo-elytrorrhaphy must be given precedence, provided there be no counter-indications. The difficulty of inverting the uterus is not an imaginary one, and it seems to me that the best way to overcome it would be to invert the uterus, placenta and all, before the placenta is separated, and between pains. This would tend to still further limit the operation to cases without extreme contraction and would bring it into rivalry with craniotomy. Its chief advantage over the Porro operation lies in not fixing the cervix several inches beyond its normal position; and here lies the germ which the author seems to be trying to develop. Should there be a condition of the uterus which would not favor the Cæsarean operation as performed by Sänger and

Leopold, should the size of the cervix or vagina render fixation of the stump in the abdominal wound too difficult, were the uterine walls not sufficiently relaxed to be inverted, or the pelvis not roomy enough to allow inversion with the placenta attached, should the condition of the tissues about the vagina and bladder contra-indicate laparo-elytrorrhaphy, and should the os dilate naturally and easily, then this operation would find its rare opportunity. The process of paring out, and inverting the cervix, as has been suggested, is easier to talk of than to perform. Any one who has seen the uterus amputated, even in cases of fibroid tumors, will agree that the loss of blood, including that taken off with the amputated pregnant uterus, and the vascularity of the stump would make the process of inverting the sliced cervix very hazardous. The stump, thus turned down, would undoubtedly shrink rapidly, and become a hard one to manage. As to opening the uterus with the cautery, I think this would not possess much advantage unless complete constriction of the uterus and broad ligaments could be made, so that bleeding would not interfere with the complete searing of the parts.

PROFESSOR BARTLETT, in closing, said: Some of the fellows taking part in the discussion, as they have stated, have not had an opportunity of hearing more of the paper than the bare proposition. Not needlessly to occupy time, I shall pass over such objections (all of which I recognize as forcible) as have been fully considered in the paper, now printed.

Dr. Jaggard refers to the authorities quoted by me as "questionable." So far as my knowledge extends, not a case cited rests upon other than unquestionable authority. The Doctor thinks the actual cautery would prove useless as a means of arresting hæmorrhage from the uterine incision.

Prior to the time of Ambrose Paré, the cautery was relied upon "to arrest all forms of hæmorrhage."

Dr. H. T. Byford has dwelt upon the difficulty of dilating the *os uteri* by artificial means, and in my opinion he has not exaggerated the difficulties often encountered in practice, where the parts are not prepared for dilation.

In regard to the embarrassment felt by the Secretary as to which operation to prefer, whether the old or the Porro method, I might say that, in face of the several substitutes and modifications, he would be amply justified in preferring the old Cæsarean section.

W. W. JAGGARD, M.D., *Editor.*

2330 Indiana Avenue, October 1886.

---

CHICAGO GYNÆCOLOGICAL SOCIETY.

